# More Frequent Clinic Visits Are Associated with Improved Outcomes for Children with NAFLD

**DOI:** 10.1155/2016/8205494

**Published:** 2016-12-12

**Authors:** Carol Lam, Robert Bandsma, Simon Ling, Marialena Mouzaki

**Affiliations:** ^1^Department of Pediatrics, Hospital for Sick Children, University of Toronto, Toronto, ON, Canada; ^2^Division of Gastroenterology, Hepatology and Nutrition, Department of Pediatrics, University of Toronto, Toronto, ON, Canada

## Abstract

*Objective*. Adult data suggest that frequent monitoring of patients with nonalcoholic fatty liver disease (NAFLD) may be associated with improved outcomes. The optimal frequency of outpatient visits for the management of pediatric NAFLD remains unknown.* Study Design*. In this retrospective study, two cohorts of patients with NAFLD, one followed on a yearly basis and one followed on 3-month intervals, were included. Both received similar advice regarding lifestyle changes. Primary outcome was change in BMI* z*-scores over a year. Secondary outcomes were the change in serum transaminases and markers of metabolic dysregulation.* Results*. Fifty-six patients were included (28 per group). The majority (71%) were male with a mean (±SD) age of 12.2 (±2.7) years. At baseline, there were no differences in BMI* z*-scores (2.8 versus 2.9; *p* = 0.72) and ALT levels (101 versus 100 U/L; *p* = 0.95) between the groups (yearly versus three-month, resp.). Twelve months later, those followed on a 3-month basis demonstrated a significant decrease in BMI (net BMI* z*-score change = −0.06; *p* = 0.37), accompanied by a significant improvement in serum ALT (−25 U/L; *p* < 0.01) and AST (−13 U/L; *p* = 0.03) levels. There were no differences in fasting lipid profiles.* Conclusion*. Frequent clinic visits are associated with improved outcomes in pediatric NAFLD.

## 1. Background

Nonalcoholic fatty liver disease (NAFLD) is the most common form of liver disease affecting children and adults in the Western world [1]. In North America, NAFLD is the second most common indication for liver transplantation in adulthood [2]. Currently, the only treatment for NAFLD is lifestyle changes (diet and exercise) that lead to a reduction in body mass index (BMI) [3]. Lifestyle changes are difficult to implement and sustain over time [4]. While behavioral interventions aimed at managing pediatric obesity have been shown to be directly proportional to the amount of time children spend with the treating team, it is not clear whether this is also true for the management of pediatric NAFLD [5]. Patients with NAFLD often represent the more extreme spectrum of obesity, as they frequently suffer from multiple other comorbidities, such as hypertension, dyslipidemia, and obstructive sleep apnea [6], suggesting that they may be a more challenging population to treat.

In a study of 924 adult patients with NAFLD, it was recently shown that weight loss, while difficult to achieve, is independently associated with the frequency of clinic visits [4]. In addition, Svetkey et al. compared two weight loss programs and showed that the more successful program mandated monthly contact between patients and their medical team [7]. Literature on this topic is limited in the pediatric population. To our knowledge, the only relevant study to-date was published by Devore et al. and it evaluated the experience of a multidisciplinary clinical program aimed at treating children with NAFLD [8]. In the program, patients were seen every 3 months. The BMI of the 39 patients who returned for follow-up, up to one year after the initial visit, had improved by a mean 0.1 standard deviation (SD) and their serum transaminase levels had decreased as well.

Considering the paucity of pediatric data on this topic we aimed at investigating whether the frequency of clinic visits impacts the outcome of patients with NAFLD. At our institution, we had the unique opportunity to address this question. Until the summer of 2012, children with NAFLD had been followed in the general hepatology clinics once a year. From that point onwards, the majority of patients were followed in a clinic dedicated to the management of children with NAFLD where they were seen every 3 months. Support by ancillary staff (e.g., dietitians and nurses) and approach for the management of these patients through implementation of lifestyle interventions (dietary improvements and introduction of exercise) otherwise remained the same.

The specific aims of this study were to

1. compare the 1-year BMI* z-*score change of those followed in the NAFLD clinic (group NAFLD3M) to an age- and gender-matched historical cohort followed in the general hepatology clinics (group HEP1Y);
2. compare the 1-year change in transaminases (ALT, AST), fasting lipid profile, and insulin resistance between groups.

We hypothesized that frequent clinic visits would be associated with improvements in the anthropometric and laboratory abnormalities of children and adolescents with NAFLD.

## 2. Patients and Methods

This was a retrospective study performed at the Hospital for Sick Children in Toronto, which included patients followed any time between 2000 and 2015. Inclusion criteria were presumed or confirmed diagnosis of NAFLD (treated as such by the medical team), followed in clinic for a minimum of 1 year, and age younger than 18 years at the end of the 1-year period. Presumed NAFLD was defined by asymptomatic elevation of ALT (×2 upper limit of normal) and echogenicity of the liver on ultrasound suggestive of fatty infiltration, accompanied by a negative work up for other liver diseases, such as viral, autoimmune, metabolic/genetic, or drug-related. Confirmed NAFLD was defined by histologic evidence of macrovesicular steatosis with or without ballooning degeneration and/or fibrosis. Exclusion criteria were the use of medications with hepatotoxic potential, metabolic conditions contributing to NAFLD (e.g., fatty acid oxidation defects, abetalipoproteinemia, lipodystrophies, panhypopituitarism, etc.), lack of clinic follow-up, and participation in other weight loss programs during the 1-year period.

Physicians and registered dietitians gave patients and families followed in both clinics similar advice regarding lifestyle changes (in fact the same dietitian worked in both clinics). Specifically, they were asked to stop consuming all sugar-sweetened beverages and to limit milk consumption to two glasses a day (fat-free milk). They were also asked to not skip meals and ensure they consumed breakfast, lunch, dinner, and two snacks between meals; however, they were advised against consuming bedtime snacks. They were instructed to consume protein with each meal and to cut back their portion sizes by 10–20%. Given the lack of consensus regarding the optimal diet for patients with NAFLD [9–11], none of the patients were asked to go on a specific diet, apart from the above. The aforementioned dietary interventions were based on healthy eating practices, as per Canada's Food Guide (http://www.hc-sc.gc.ca/fn-an/food-guide-aliment/index-eng.php) and the available literature to date [12, 13]. None of the patients were given handouts or other documents to assist with the implementation of the suggested lifestyle changes. At follow-up, patients met with the physicians; there were no visits organized to meet specifically with dietitian or nurse only in either hepatology or NAFLD clinics.

Consecutive patients followed on a 3-month basis in the NAFLD clinics, who met inclusion and exclusion criteria, were included initially. Data from age and gender matched patients followed on an annual basis were subsequently included to form the comparative group. For the purposes of the study, within the 1-year period, the first clinic visit was considered the baseline visit, whereas the visit closest to 12 months following the baseline was the outcome visit. Research Ethics Board approval was obtained prior to the retrospective chart review. From here on in this manuscript, children followed every 3 months in the NAFLD clinic are referred to as “NAFLD3M” and children followed for at least 1 year by the general hepatology clinics are referred to as “HEP1Y.”

### 2.1. Data Collection

Clinical data collected included the patients' gender, ethnicity, liver biopsy results, age at baseline visit, weight, height, and BMI* z-*scores at both baseline and outcome visits. Laboratory data collected at both time-points were ALT, AST, *γ*-glutamyl transferase (GGT), alkaline phosphatase (ALP), fasting triglycerides, total cholesterol, low-density lipoprotein (LDL), high-density lipoprotein (HDL), glucose, insulin, and HbA1c. HOMA-IR was calculated as per standard clinical practice [14].

### 2.2. Statistical Analyses

Continuous variables were compared using the Student's* t*-test (paired when indicated) or the Wilcoxon rank-sum (Mann-Whitney) test in cases where the data were not normally distributed. *χ* ^2^ square testing was used to compare categorical variables. The software used for the statistical analyses was StataMP 13.0 for Mac. As a similar study had not been done in children or adults previously, we based our sample size calculation on the study by Devore et al. [8]. In order to identify a difference in the change in BMI* z-*score by 0.2 between the groups with a power of 0.9 and a significance threshold set at 0.05 and assuming a SD of 0.2 [8], 22 patients would have to be included in each arm. We aimed at including 28 patients in each group to maximize our power, in case the actual SD was higher than 0.2.

## 3. Results

Fifty-six patients were included in the study; 28 followed in the HEP1Y and 28 in the NAFLD3M clinic. The median number of visits to the NAFLD3M clinic in the year was 5 (including baseline and outcome visit), with a minimum of 3 visits (*n* = 5 patients) and a maximum of 6 visits (*n* = 2). Of the 56 patients included in the study cohort, 15 had had a liver biopsy (12 from group NAFLD3M and 3 from group HEP1Y), which had showed NASH in 13 and nonalcoholic fatty liver (NAFL) in 2. Both patients with NAFL were from group NAFLD3M. The baseline characteristics of the patients are shown in Table 1.

Patients included in this study were on average young teenagers, with an age range of 6–16 years. The proportion of Hispanics was not statistically different between the groups (*p* = 0.48). At baseline, the mean weight of group NAFLD3M was 76 kg (±19 kg) and that of group HEP1Y was 85 kg (±31 kg; *p* = 0.54). Similarly, the heights of both groups of patients were not different (158 cm ± 14 cm versus 156 cm ± 22 cm, *p* = 0.95). The mean BMI* z-*score of the entire cohort was 2.8 (±0.7), without significant differences between the groups (NAFLD3M* z-*score = 2.9 versus HEP1Y* z-*score = 2.8; *p* = 0.72). The BMI* z-*score was >2 in 89% of patients in group NAFLD3M and 82% of those in group HEP1Y (*p* = 0.44). None of the patients were treated with vitamin E.

At the outcome visit, those followed on a 3-month basis had a significant improvement in BMI* z-*score compared to baseline (−0.25; *p* < 0.01), as opposed to those followed annually, whose BMI* z-*score had remained essentially unchanged (−0.06; *p* = 0.37). The mean change in BMI between visits of those followed in the NAFLD clinic was −0.14 kg/m^2^ (range: −9.6 to 3.6 kg/m^2^). Despite the improvement, a significant proportion of patients in both groups continued to have a BMI* z-*score >2 at the outcome visit (75% of those in group NAFLD3M and 81% of those in group HEP1Y; *p* = 0.61). None of the patients followed on a yearly basis and only two patients followed in 3 monthly intervals normalized their BMI* z-*scores (<1 SD).

The baseline laboratory investigations were not different between the NAFLD3M and HEP1Y groups: ALT 100 versus 101 (*p* = 0.95); AST 57 versus 60 (*p* = 0.69); GGT 41 versus 63 (*p* = 0.17); and ALP 247 versus 238 (*p* = 0.81). At the outcome visit, the serum transaminases improved significantly in patients followed every 3 months (mean ALT change: −25 U/L, *p* = 0.02; mean AST change: −13 U/L, *p* = 0.03; Table 2) compared to baseline. Those who were followed on a yearly basis did not have a significant improvement in their transaminases (Table 2). The number of patients whose ALT normalized at 1 year was not different between the groups (17% versus 4%, *p* = 0.32; in those followed in the NAFLD3M and HEP1Y clinics, resp.). There were no significant changes in lipid profiles over time between either group, nor where there any significant differences in platelet count. There were limited data available for fasting glucose and insulin levels, preventing the calculation of HOMA-IR or further statistical analyses.

## 4. Discussion

This study shows that in a cohort of young children and adolescents with NAFLD increased frequency of clinic visits is associated with a significant improvement in BMI* z-*scores, accompanied by a significant decrease in serum transaminase levels over a period of 12 months. Despite these improvements, lipid profiles did not improve over time in either group.

This is the first pediatric study that addresses the impact of clinic visit frequency on the success of implementing lifestyle changes, as indicated by changes in BMI* z-*scores, for the management of NAFLD. The results of this study are in agreement with adult data showing that clinic visit frequency is a predictor of weight loss in patients with NAFLD [4, 15]. In children, Devore et al. recently reported the outcomes of a multidisciplinary clinical program caring for patients with NAFLD at a university hospital [8]. The frequency of monitoring was every 3 months; however, only 47% of the 108 patients who were followed in the clinic returned for a 1-year visit. The authors reported a small (−0.1) improvement in the BMI* z-*scores of patients who committed to 3-month visits, which, similar to our results, was accompanied by decreased transaminase levels. The outcomes of those who did not comply with the program were not known, and, hence, it is not possible to draw conclusions about superiority of the 3-month visits from Devore et al.'s study.

Research in the field of obesity suggests that while subjects can lose weight successfully by participating in various lifestyle intervention programs, they tend to regain the weight over time [16, 17]. Frequent face-to-face contact between patients and treating teams has been shown to be superior to other approaches, such as self-directed or internet-based support, for weight maintenance following weight loss [7, 18]. While there are no studies specifically addressing this question in patients with NAFLD, the aforementioned data suggest that patients require ongoing encouragement and support to sustain lifestyle changes that they have implemented early on. Considering the improved outcomes of children followed on a 3-month basis in our study, and the need for ongoing weight management in these patients, it would be sensible to maintain more frequent clinic visits for patients who have not reached a healthy BMI.

Three-month visits may be difficult to accommodate in a busy subspecialty clinic. As opposed to weight loss programs, where patients may be followed by a multitude of practitioners (dietitians, psychologists, exercise physiologists, etc.) and may be asked to follow predetermined diets, the lifestyle advice given to children in the NAFLD and general hepatology clinics was focused on portion control and substitution of foods and beverages by lower calorie alternatives. This is something that can also be achieved in a clinic visit with a family doctor or general pediatrician. As a result, combined efforts to follow children with NAFLD closely by both primary care providers and gastroenterologists/hepatologists may lead to improved outcomes without substantially increasing the burden on the subspecialty clinics. It is not clear whether other approaches, such as frequent phone calls or telemedicine type of interactions, could also serve as a means of providing frequent follow-up for these patients successfully. This would need to be investigated in future randomized controlled trials.

In our study, the BMI* z-*scores of patients who were followed on a three-month basis decreased by −0.25 SD, on average. This level of improvement, which is equivalent to ~2.5% decrease in BMI percentile, may not appear to be clinically significant; however, it was associated with an improvement in serum transaminase levels. Recently, Vilar-Gomez et al. reported the histological changes seen with weight loss in adult patients with NASH [3]. In that study, weight loss >5% was associated with resolution of NASH in more than half of the patients in the study (58%), while weight loss of ≥10% was associated with fibrosis improvement in 45% and resolution of NASH in 90% of patients [3]. A BMI* z-*score improvement of −0.25 SD (assuming a stable height) corresponds to an approximate weight loss of 6%. Liver biopsy data were not available in our study to assess the histological progression of the fatty liver disease over the 12-month period; and, hence, we cannot confirm that these results suggest a histological improvement in those followed every 3 months. However, given the adult literature, it is possible that the combination of reduced BMI and ALT are associated with histological improvement. This remains to be investigated further.

In our institution, patients received the same support by health professionals, including nurses and clinical dietitians, in order to achieve a healthier lifestyle, regardless of which clinic they were followed in (i.e., the NAFLD or general hepatology clinics). In addition, patients' baseline characteristics were strikingly similar, as shown by their almost identical baseline BMI* z-*scores and serum ALT levels. This suggests that the improved outcomes seen in group NAFLD3M are likely to be related to the frequency of outpatient clinic follow-up and not secondary to sampling differences. It is possible however that different physician approaches between the dedicated NAFLD and general hepatology clinics may have also had an impact on patient outcomes. The differences in overall management (e.g., frequency of liver biopsy use to confirm the diagnosis) may have been personal (different physicians) or the result of the different eras of the two clinics (before and after 2012). This can be investigated further in larger studies where patients are randomized to outpatient clinic monitoring of differing frequencies.

It is important to note that at the outcome visit, 75% of those in group NAFLD3M and 81% of those in group HEP1Y still retained a BMI* z-*score that was higher than 2 (BMI > 97th percentile), indicating that these patients were still within the obesity range. This finding is in agreement with the report by Dudekula et al., which demonstrated that the majority of patients with NAFLD struggle to lose weight, with only 19.8% of those who partook in a weight loss program losing ≥5% of their baseline weight [4]. In addition, in our study, only 2 patients in the group were followed on a 3-month basis and none of those followed annually normalized their BMI. These results suggest that lifestyle interventions have a limited impact on the natural history of patients with NAFLD and underscore the urgent need for novel treatment approaches, medical or surgical, to allow for more patients to reach a healthier BMI.

Limitations of this study include its retrospective nature and the lack of data on histological progression. This study also lacked data from noninvasive assessments of hepatic fibrosis (e.g., transient elastography or MR elastography), as these have only recently become available at our institution. Timeline bias may have affected the results of this study, as the NAFLD clinic was created immediately following the release of new guidelines [19]. However, the pediatric section of these guidelines was limited and unlikely to have changed clinical practice significantly. In addition, there were no available data on body composition and hence we could only assess changes in BMI* z-*scores. However, considering that weight loss has been associated with improvement in NAFLD liver histology, it is predicted that the severity of liver injury progressed less in those followed more frequently. Lastly, over the 12-month study period, we had access to limited paired data to assess the progression of insulin sensitivity/type 2 diabetes mellitus and to correlate these results with clinic visit frequency.

## 5. Conclusion

To conclude, frequent monitoring is associated with improved outcomes in pediatric patients with NAFLD. While changes in BMI* z-*scores may appear small, their association with improved serum transaminase levels suggests a clinically significant improvement. Considering the importance of frequent monitoring for weight maintenance following successful implementation of weight loss programs, it would appear that these patients need close and frequent follow-up on a long-term basis to ensure ongoing success.

## Figures and Tables

**Table 1:** Demographics and baseline patient characteristics.

Variable	Followed every 3 months (Group NAFLD3M)	Followed annually (Group HEP1Y)	*p* value
Age in years	12.3 (2.4)	12.0 (2.7)	0.72
Gender (% male)	71%	71%	1.00
Ethnicity/race; *n* (%)			
Caucasian	19 (68%)	10 (36%)	0.28
Asian	6 (21%)	6 (21%)	
Hispanic	2 (7%)	7 (25%)	
Unknown	1 (4%)	5 (18%)	
Family history of NAFLD/NASH (%)	8 (29%)	7 (25%)	0.55
Outpatient clinic visits during one year of follow-up; *n* (SD)	2.4 (0.8)	0.1 (0.0)	<0.01
Months between baseline and outcome visits; *n* (SD)	12.3 (2.6)	12.0 (2.2)	0.63

Values are expressed as means (SD).

**Table 2:** Change in laboratory values between baseline and outcome clinic visits.

	Group	Baseline	Outcome	*p* value
ALT (U/L)	NAFLD3M	100 (90)	75 (54)	0.02
	HEP1Y	101 (81)	85 (70)	0.09
AST (U/L)	NAFLD3M	57 (43)	44 (27)	0.03
	HEP1Y	60 (35)	52 (40)	0.11
GGT (U/L)	NAFLD3M	41 (30)	31 (18)	<0.01
	HEP1Y	63 (53)	57 (48)	0.07
ALP (U/L)	NAFLD3M	247 (107)	206 (89)	<0.01
	HEP1Y	238 (95)	212 (96)	0.05
LDL (mmol/L)	NAFLD3M	2.5 (0.6)	2.5 (0.6)	0.38
	HEP1Y	2.9 (0.1)	2.7 (0.3)	0.19
HDL (mmol/L)	NAFLD3M	1.2 (0.3)	1.1 (0.3)	0.36
	HEP1Y	0.9 (0.1)	0.9 (0.1)	0.25
Total cholesterol (mmol/L)	NAFLD3M	4.4 (0.8)	4.3 (0.7)	0.19
	HEP1Y	5.2 (1.1)	5.0 (1.3)	0.53
Triglycerides (mmol/L)	NAFLD3M	1.3 (0.8)	1.4 (1.0)	0.56
	HEP1Y	2.1 (1.3)	1.7 (1.0)	0.37
PLT (×10^9^/L)	NAFLD3M	288 (49)	290 (62)	0.88
	HEP1Y	326 (55)	316 (56)	0.33

Data are expressed as means (SD).

## Abbreviations

ALP: Alkaline phosphatase
ALT: Alanine aminotransferase
AST: Aspartate aminotransferase
BMI: Body mass index
GGT: Gamma-glutamyl transferase
HbA1c: Hemoglobin A1C
HDL: High-density lipoprotein
HOMA-IR: Homeostatic model assessment-insulin resistance
LDL: Low-density lipoprotein
NAFLD: Nonalcoholic fatty liver disease
NASH: Nonalcoholic steatohepatitis
SD: Standard deviation.

## Additional Points

*What Is Known on This Subject*. It is known that NAFLD is challenging to manage; its mainstay of treatment is lifestyle changes, such as diet and exercise. There are currently no data to support recommendations regarding the optimal frequency of outpatient monitoring of children with NAFLD.* What This Study Adds*. The authors show that more frequent outpatient clinic visits are associated with improvements in BMI and ALT levels in children with NAFLD. This information is crucial for both subspecialists and general practitioners, as together they can provide this intensity of monitoring.

## References

[1] Loomba R., Sanyal A. J. (2013). The global NAFLD epidemic. *Nature Reviews Gastroenterology and Hepatology*.

[2] Wong R. J., Aguilar M., Cheung R. (2015). Nonalcoholic steatohepatitis is the second leading etiology of liver disease among adults awaiting liver transplantation in the United States. *Gastroenterology*.

[3] Vilar-Gomez E., Martinez-Perez Y., Calzadilla-Bertot L. (2015). Weight loss through lifestyle modification significantly reduces features of nonalcoholic steatohepatitis. *Gastroenterology*.

[4] Dudekula A., Rachakonda V., Shaik B., Behari J. (2014). Weight loss in nonalcoholic fatty liver disease patients in an ambulatory care setting is largely unsuccessful but correlates with frequency of clinic visits. *PLoS ONE*.

[5] Whitlock E. P., O'Connor E. A., Williams S. B., Beil T. L., Lutz K. W. (2010). Effectiveness of weight management interventions in children: a targeted systematic review for the USPSTF. *Pediatrics*.

[6] Nobili V., Cutrera R., Liccardo D. (2014). Obstructive sleep apnea syndrome affects liver histology and inflammatory cell activation in pediatric nonalcoholic fatty liver disease, regardless of obesity/insulin resistance. *American Journal of Respiratory and Critical Care Medicine*.

[7] Svetkey L. P., Stevens V. J., Brantley P. J. (2008). Comparison of strategies for sustaining weight loss: the weight loss maintenance randomized controlled trial. *The Journal of the American Medical Association*.

[8] Devore S., Kohli R., Lake K. (2013). A multidisciplinary clinical program is effective in stabilizing BMI and reducing transaminase levels in pediatric patients with NAFLD. *Journal of Pediatric Gastroenterology and Nutrition*.

[9] Nobili V., Svegliati-Baroni G., Alisi A., Miele L., Valenti L., Vajro P. (2013). A 360-degree overview of paediatric NAFLD: recent insights. *Journal of Hepatology*.

[10] Finelli C., Tarantino G. (2012). Is there any consensus as to what diet or lifestyle approach is the right one for NAFLD patients?. *Journal of Gastrointestinal and Liver Diseases*.

[11] Rusu E., Enache G., Jinga M. (2015). Medical nutrition therapy in non-alcoholic fatty liver disease—a review of literature. *Journal of Medicine and Life*.

[12] Ma J., Fox C. S., Jacques P. F. (2015). Sugar-sweetened beverage, diet soda, and fatty liver disease in the Framingham Heart Study cohorts. *Journal of Hepatology*.

[13] Jin R., Le N.-A., Liu S. (2012). Children with NAFLD are more sensitive to the adverse metabolic effects of fructose beverages than children without NAFLD. *Journal of Clinical Endocrinology and Metabolism*.

[14] Keskin M., Kurtoglu S., Kendirci M., Atabek M. E., Yazici C. (2005). Homeostasis model assessment is more reliable than the fasting glucose/insulin ratio and quantitative insulin sensitivity check index for assessing insulin resistance among obese children and adolescents. *Pediatrics*.

[15] George A. St., Bauman A., Johnston A., Farrell G., Chey T., George J. (2009). Effect of a lifestyle intervention in patients with abnormal liver enzymes and metabolic risk factors. *Journal of Gastroenterology and Hepatology*.

[16] Aller E. E. J. G., Larsen T. M., Claus H. (2014). Weight loss maintenance in overweight subjects on ad libitum diets with high or low protein content and glycemic index: the DIOGENES trial 12-month results. *International Journal of Obesity*.

[17] Larsen T. M., Dalskov S.-M., Van Baak M. (2010). Diets with high or low protein content and glycemic index for weight-loss maintenance. *New England Journal of Medicine*.

[18] Wing R. R., Tate D. F., Gorin A. A., Raynor H. A., Fava J. L. (2006). A self-regulation program for maintenance of weight loss. *The New England Journal of Medicine*.

[19] Chalasani N., Younossi Z., Lavine J. E. (2012). The diagnosis and management of non-alcoholic fatty liver disease: practice guideline by the american association for the study of liver diseases, american college of gastroenterology, and the american gastroenterological association. *Hepatology*.

